# Umbilical Cord Blood and Cord Tissue-Derived Cell Therapies for Neonatal Morbidities: Current Status and Future Challenges

**DOI:** 10.1093/stcltm/szab024

**Published:** 2022-03-08

**Authors:** Lindsay Zhou, Courtney McDonald, Tamara Yawno, Graham Jenkin, Suzanne Miller, Atul Malhotra

**Affiliations:** The Ritchie Centre, Hudson Institute of Medical Research, Clayton, VIC, Australia; Department of Paediatrics, Monash University, Clayton, VIC, Australia; Monash Children’s Hospital, Monash Health, Clayton, VIC, Australia; The Ritchie Centre, Hudson Institute of Medical Research, Clayton, VIC, Australia; Department of Obstetrics and Gynaecology, Monash University, Clayton, VIC, Australia; The Ritchie Centre, Hudson Institute of Medical Research, Clayton, VIC, Australia; Department of Paediatrics, Monash University, Clayton, VIC, Australia; Department of Obstetrics and Gynaecology, Monash University, Clayton, VIC, Australia; The Ritchie Centre, Hudson Institute of Medical Research, Clayton, VIC, Australia; Department of Obstetrics and Gynaecology, Monash University, Clayton, VIC, Australia; The Ritchie Centre, Hudson Institute of Medical Research, Clayton, VIC, Australia; Department of Obstetrics and Gynaecology, Monash University, Clayton, VIC, Australia; The Ritchie Centre, Hudson Institute of Medical Research, Clayton, VIC, Australia; Department of Paediatrics, Monash University, Clayton, VIC, Australia; Monash Children’s Hospital, Monash Health, Clayton, VIC, Australia

**Keywords:** umbilical cord blood stem cells, umbilical cord tissue-derived cells, cell therapy

## Abstract

Cell therapies are an emerging focus for neonatal research, with benefits documented for neonatal respiratory, neurological, and cardiac conditions in pre-clinical studies. Umbilical cord blood (UCB) and umbilical cord (UC) tissue-derived cell therapy is particularly appealing for preventative or regenerative treatment of neonatal morbidities; they are a resource that can be collected at birth and used as an autologous or allogeneic therapy. Moreover, UCB contains a diverse mix of stem and progenitor cells that demonstrate paracrine actions to mitigate damaging inflammatory, immune, oxidative stress, and cell death pathways in several organ systems. In the past decade, published results from early-phase clinical studies have explored the use of these cells as a therapeutic intervention in neonates. We present a systematic review of published and registered clinical trials of UCB and cord tissue-derived cell therapies for neonatal morbidities. This search yielded 12 completed clinical studies: 7 were open-label phase I and II safety and feasibility trials, 3 were open-label dose-escalation trials, 1 was a open-label placebo-controlled trial, and 1 was a phase II randomized controlled trial. Participants totaled 206 infants worldwide; 123 (60%) were full-term infants and 83 (40%) were preterm. A majority (64.5%) received cells via an intravenous route; however, 54 (26.2%) received cells via intratracheal administration, 10 (4.8%) intraoperative cardiac injection, and 9 (4.3%) by direct intraventricular (brain) injection. Assessment of efficacy to date is limited given completed studies have principally been phase I and II safety studies. A further 24 trials investigating UCB and UC-derived cell therapies in neonates are currently registered.

## Graphical Abstract

**Graphical Abstract:**
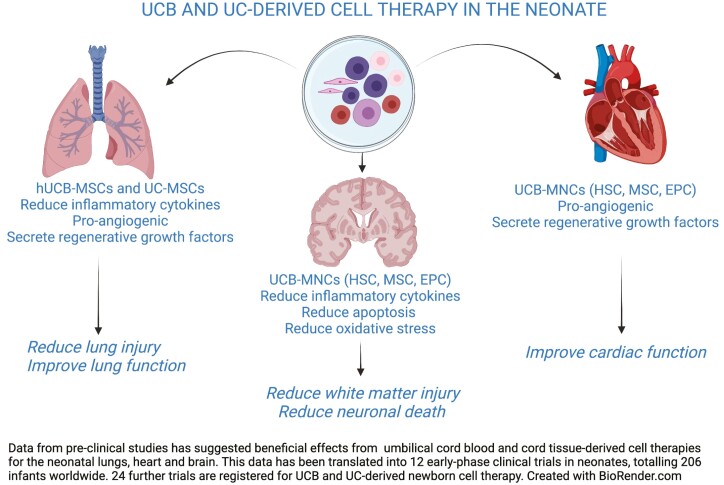

**Significance Statement**

Umbilical cord blood and cord tissue-derived cell therapies are an emerging area of translational research in neonatal medicine. This concise review provides a systematic review of completed clinical trials of UCB and cord tissue-derived cell therapy in neonates, describes immune and paracrine mechanisms by which these therapies may provide benefit as demonstrated by pre-clinical data, and presents future directions and challenges facing the use of these cell therapies in the neonatal population.

## Introduction

Neonatal morbidities affect a significant population, with conditions, including prematurity, birth asphyxia, chorioamnionitis, fetal growth restriction, and congenital malformations.^1^ While advances in perinatal care have improved survival rates for sick neonates over the last 50 years, there remains a paucity of targeted interventions to reduce chronic morbidity experienced by this population, in particular effects on the lungs, heart, and/or brain.^2-4^ Cell therapy is an emerging field of regenerative medicine in neonatology, and translation from pre-clinical evidence to early-phase clinical trials is underway.

Umbilical cord blood (UCB) can be collected at both vaginal and cesarean deliveries, and the mononuclear cell fraction contains a rich mix of stem and progenitor cells that separately and combined have therapeutic potential.^5-7^ Similarly, umbilical cord (UC) tissue can be collected, stored, and used as a source of cultured mesenchymal stem cells (UC-MSCs).^8,9^

In this concise review, we examine published clinical studies and registered clinical trials of UCB and UC-derived cell therapy for neonatal morbidities. In doing so, we have defined the major neonatal disease targets of lung, heart, and brain pathologies. Accordingly, we discuss the published clinical studies for each of those target diseases and discuss likely mechanism(s) of action of these cells from pre-clinical data. Finally, we discuss the challenges and potential future directions for UCB and UC-derived cell therapy in neonates that must be addressed to inform design future trial design.

### Umbilical Cord Blood Cells

UCB has been used as a source of stem and progenitor cells since its first use for hematopoietic stem cell transplantation for Fanconi anemia in 1988^10^ and is now widely applied in the treatment of hematological malignancies and other conditions requiring stem cell transplantation.^11^ However, the immune-modulatory and neuroprotective effects of UCB and UC-derived cell populations in the absence of engraftment are an increasing area of study and a target for therapeutic investigation in clinical trials.

UCB can be collected in high volumes, yielding on average 81 mL per collection in term infants providing a nucleated cell count of 3.89-15.68 × 10^8^ cells.^12,13^ Even in preterm infants, there is evidence that UCB can be collected in adequate volumes for cell therapy.^14^

The UCB mononuclear fraction contains a wide range of mature and stem/progenitor cells, which have been shown to have a broad range of differentiation potential.^15-18^ These populations include hematopoietic stem cells (HSCs), mesenchymal stromal cells (MSCs), endothelial progenitor cells (EPCs), T cells, natural killer (NK) cells, dendritic cells, regulatory T cells, monocyte-derived suppressor cells (MDSCs), and unrestricted somatic stem cells (USSCs) which combined as the mononuclear cell fraction, or as separate populations, have a variety of beneficial paracrine effects.^19-21^ Cultured UCB mononuclear cells (UCB-MNCs) have been shown to secrete cytokines and growth factors, such as vascular endothelial growth factor (VEGF) and anti-inflammatory cytokines like IL-10,^22^ and UCB-derived HSCs are shown to improve neurogenesis following ischemic injury via promotion of angiogenesis.^23,24^ MSCs also secrete IL-10 and VEGF, as well as brain-derived neurotrophic factor, which play a vital role in development, neuroprotection, and regeneration by suppressing brain inflammation and promoting angiogenesis.^19,25^ EPCs also have the ability to mediate the neovascularization process, demonstrated in vitro and in vivo via promotion of angiogenesis using animal models of tissue injury.^26-28^ USSCs have been isolated and used in pre-clinical models of brain injury, showing improved microstructure, reduced inflammation, and improved neurobehavioral outcomes in a rabbit model of intraventricular hemorrhage.^29,30^

The above cell types are all included when UCB-MNCs are given in trials as a UCB cell therapy; however, the cell content and ratio of cell types can vary with gestational age and context of delivery, including an increased proportion of CD34^+^ cells in the preterm population.^31,32^ Other studies have administered UCB and cord-derived MSCs (hUCB-MSCs and UC-MSCs), wherein MSCs are isolated and expanded from UCB or cord tissue.

**Figure 1.**
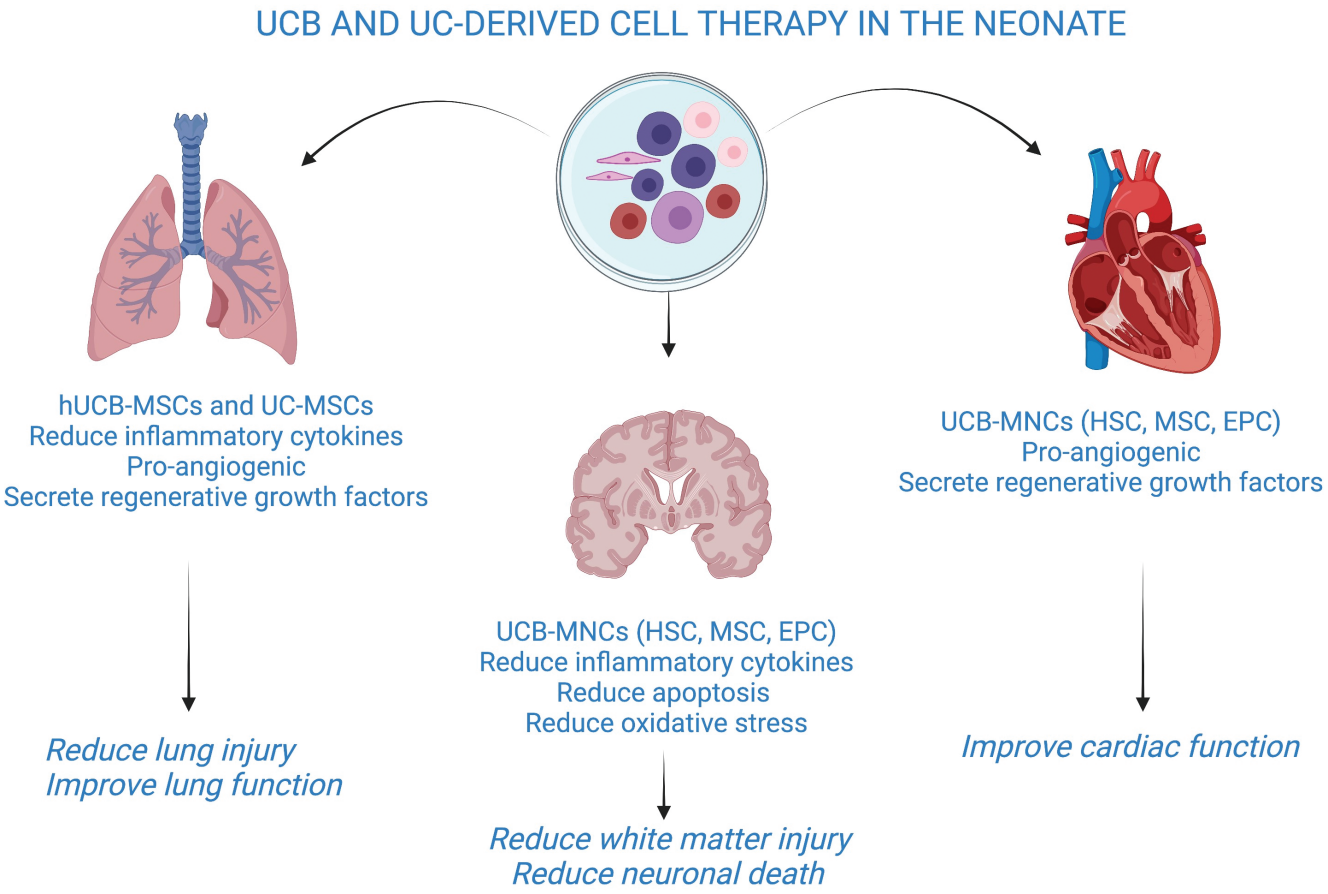
Umbilical cord blood and cord tissue-derived cell therapy in the neonate. Created with BioRender.com.

A diagram demonstrating UCB and UC-derived cells as a targeted intervention for neonatal morbidities and their ­suggested benefits from pre-clinical studies is shown in Fig. 1.

## A Systematic Review of Published Clinical Trials Using UCB and Cord Tissue-Derived Cell Therapies for Neonatal Morbidities

We conducted a systematic search of PubMed, Ovid Medline, Google Scholar, ClinicalTrials.gov, the Australia New Zealand (anzctr.org.au), International (www.who.int), European (clinicaltrialsregister.eu), and Chinese (chictr.org.cn) Clinical Trial Registries as well as the Cochrane Controlled Register of Trials and CellTrials.org for studies administering UCB and UC-derived cell therapies to neonates. The initial search was conducted in November 2020 and updated in July 2021. Search terms included “umbilical cord blood,” “umbilical cord blood stem cells,” “mesenchymal stem cells,” “mesenchymal stromal cells,” “cord blood,” “infant,” “neonate,” and “newborn.” Trial registries were searched with these terms as well as “bronchopulmonary dysplasia,” “congenital heart disease,” “hypoxic ischemic encephalopathy,” and “preterm brain injury” to capture all neonatal cell therapy studies. Studies were included if they used UCB-derived and UC-derived cell therapies for disorders diagnosed in the neonatal period (<1 month of age). Studies using UCB cells to conduct hematopoietic stem cell transplantation for conditions, such as inborn errors of metabolism and hematological malignancy, and replacement of red cells for anemia, were excluded. Studies were also excluded if the UCB cells were administered to children for disorders detected after 1 month of age, such as established cerebral palsy or diabetes mellitus. The search strategy was adapted from the Cochrane Neonatal standard search strategy, with PRISMA flow chart listed in Supplementary Appendix 1.

### Results—Published Clinical Trials of UCB and UC-Derived Cell Therapies for Neonatal Morbidities

After exclusions, our search yielded 12 studies describing the administration of UCB and UC-derived cell therapies for neonatal morbidities, which included a total of 206 participants worldwide. Seven were open-label phase I safety and feasibility trials, 3 open-label dose-escalation trials, 1 open-label placebo control trial, and 1 phase II randomized controlled trial (RCT).

Of the 206 trial participants, at the time of treatment 123 (60%) were term infants (37 weeks completed gestational age or greater) and 83 (40%) were preterm (<37 weeks completed gestational age. A majority (64.5%) of infants received cells via the intravenous route; however, 54 (26.2%) received cells via intratracheal administration, 10 (4.8%) by intraoperative cardiac injection, and 9 (4.3%) by direct intraventricular (brain) injection.

**Figure 2.**
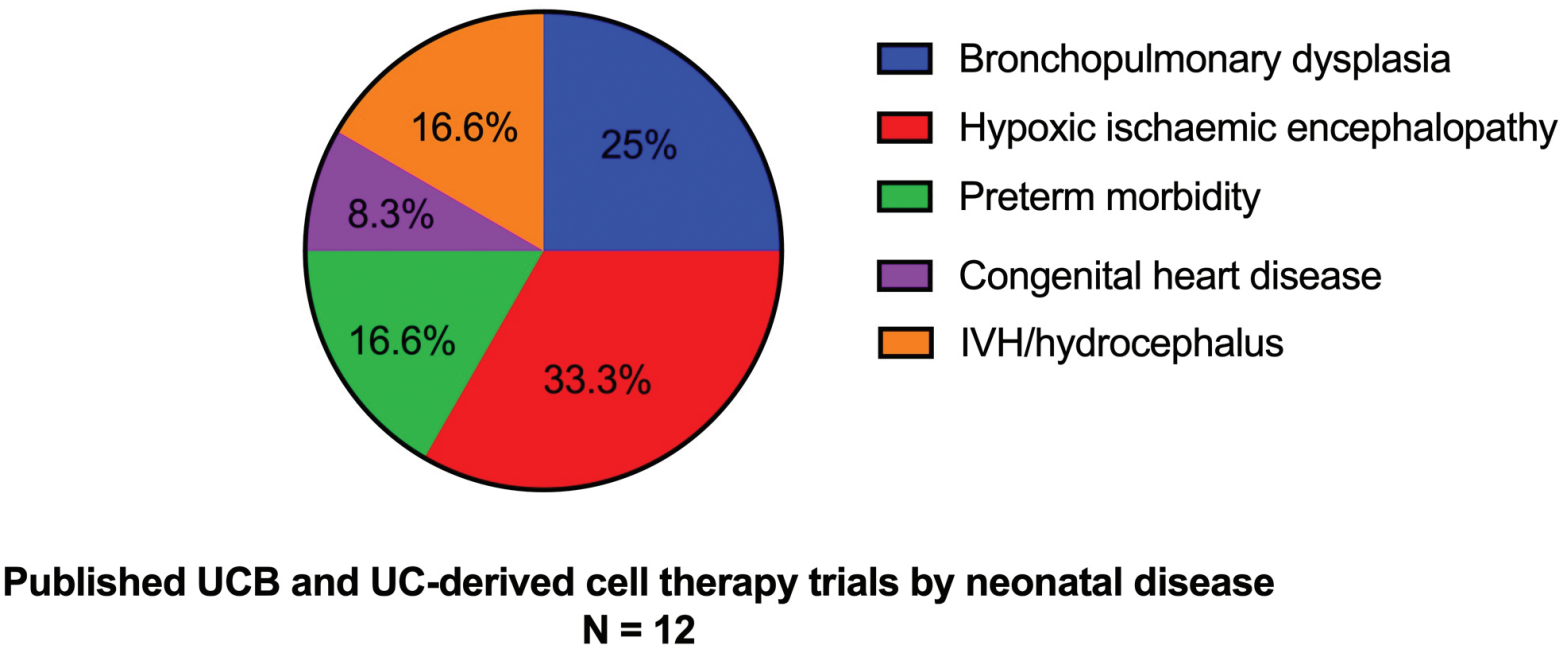
Published UCB and UC-derived cell therapy trials by neonatal morbidity. Abbreviations: UC, umbilical cord; UCB, umbilical cord blood.

A summary of published UCB and UC-derived cell therapy trials for neonatal morbidities is listed in Table 1, and the disease of interest for those trials is shown in Fig. 2.

**Table 1.** Completed UCB and UC-derived cell therapy trials in neonates.

Study	Location	Design	Population	Intervention	Primary outcome
Anh et al, 2021 https://doi.org/10.1002/sctm.20-0330	South Korea	Phase II randomized controlled trial	Preterm infants 23-28 weeks at risk of BPD *n* = 33	Intratracheal allogeneic UC-MSCs 1 × 10^7^/kg days 5-14	Death or moderate-severe BPD
Cotten et al, 2020 https://doi.org/10.1016/j.jcyt.2020.04.052	US	Open-label, phase I	Term infants with HIE *n* = 6	Intravenous allogeneic UC-MSCs 2 × 10^6^/kg at 48 hours and 2 months	Safety and feasibility within 2 weeks of administration
Tsuji et al, 2020 https://doi.org/10.1038/s41598-020-61311-9	Japan	Open-label, phase I	Term infants with HIE *n* = 6	Intravenous autologous UC-MNCs 1.4-10.9 × 10^8^ at 24, 48, and 72 hours	Safety and feasibility until 18 months
Powell et al, 2019 https://doi.org/10.1016/j.jpeds.2019.02.029	US	Open-label, phase I dose-escalation	Preterm infants <28 weeks at risk of BPD *n* = 12	Intratracheal allogeneic UC-MSCs 1-2 × 10^7^/kg days 5-14	Safety within 84 days of administration
Ren et al, 2019 https://doi.org/10.1002/sctm.19-0106	China	Open-label, phase I placebo-control	Preterm infants <35 weeks *n* = 15	Intravenous autologous UC-MNCs 5 × 10^7^/kg day 1	Mortality before discharge, rate of preterm complications
Burkhart et al, 2019 https://doi.org/10.1016/j.jtcvs.2019.06.001	US	Open-label, phase I	Term infants hypoplastic left heart syndrome *n* = 10	Intracardiac autologous UC-MNCs 1-3 × 10^6^/kg during surgery	Safety and feasibility during surgery and until 6 months
Anh et al, 2018 https://doi.org/10.1002/sctm.17-0219	South Korea	Open-label, phase I dose-escalation	Preterm infants with severe IVH *n* = 9	Intraventricular injection of allogeneic UC-MSCs 1-5 × 10^6^/kg days 7-15	Safety and feasibility until term-corrected age
Kotowski et al, 2017 PMID: 29550804	Poland	Open-label, phase I with matched controls	Preterm infants <32 weeks *n* = 5	Intravenous whole UCB on day 5	Safety and feasibility within 14 days
Sun et al, 2015 https://doi.org/10.1038/pr.2015.161	US	Open-label, phase I	Term infants with congenital hydrocephalus *n* = 76	Intravenous autologous UC-MNCs 1-5 × 10^7^/kg at day 6-4.5 years (median 2 months)	Safety and feasibility until 12 months
Cotten et al, 2014 https://doi.org/10.1016/j.jpeds.2013.11.036	US	Open-label, phase I, matched controls	Term infants with HIE *n* = 23	Intravenous autologous UC-MNCs 1-5 × 10^7^/kg 12, 24, 48, and 72 hours	Safety and feasibility until 12 months
Chang et al, 2014 https://doi.org/10.1016/j.jpeds.2013.12.011	South Korea	Open-label, phase I dose-escalation	Preterm infants 23-29 weeks at risk of BPD *n* = 9	Intratracheal allogeneic UC-MSCs 1-2 × 10^7^/kg days 7-14	Safety until term-corrected age
Jiun et al, 2013 https://doi.org/10.22494/cot.v1i1.45	Singapore	Open-label, phase I	Term infants with HIE *n* = 2	Intravenous autologous UC-MNCs 6 × 10^6^ at <24, 24, 48, and 72 hours	Safety and feasibility until 12 months

Abbreviations: BPD, bronchopulmonary dysplasia; HIE, hypoxic ischemic encephalopathy; IVH, intraventricular hemorrhage; UC, umbilical cord; UCB, umbilical cord blood; UC-MNCs, umbilical cord-derived mononuclear cells; UC-MSCs, umbilical cord-derived mesenchymal stem/stromal cells.

### Ongoing Registered Clinical Trials of UCB and UC-Derived Cell Therapies in Neonates

In addition to published trials, our search yielded 24 registered, not yet completed clinical trials of UCB and UC-derived cell therapies in neonates in 11 different countries, with 13 of those listed as actively recruiting as of July 2021. Seventy-five percent of registered trials targeted bronchopulmonary dysplasia (*n* = 9) and term hypoxic ischemic encephalopathy (*n* = 9), followed by congenital heart disease (*n* = 3), preterm brain injury (*n* = 2), and 1 study administering autologous UCB-MNCs to infants with congenital diaphragmatic hernia.

**Figure 3.**
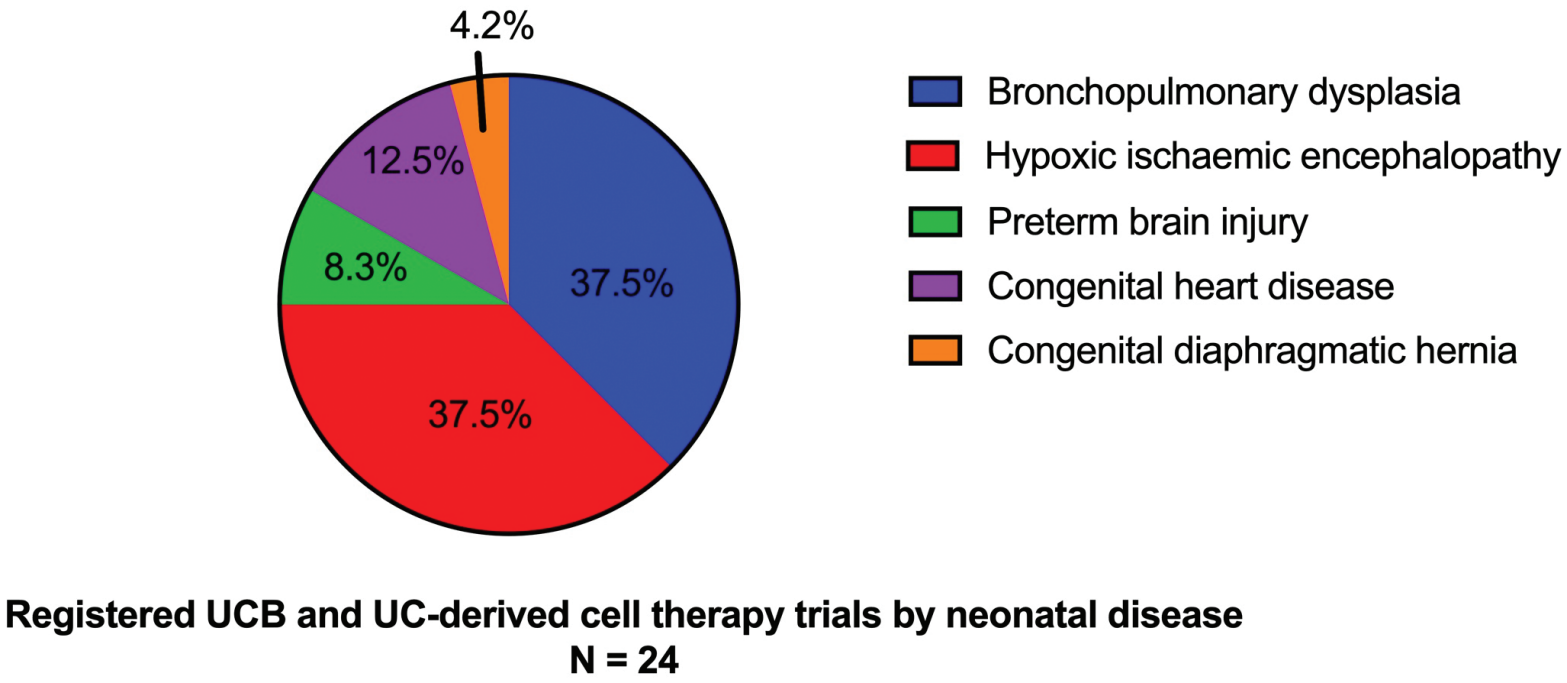
Registered, not completed UCB and UC-derived cell therapy trials by neonatal morbidity. Abbreviations: UC, umbilical cord; UCB, umbilical cord blood.

**Figure 4.**
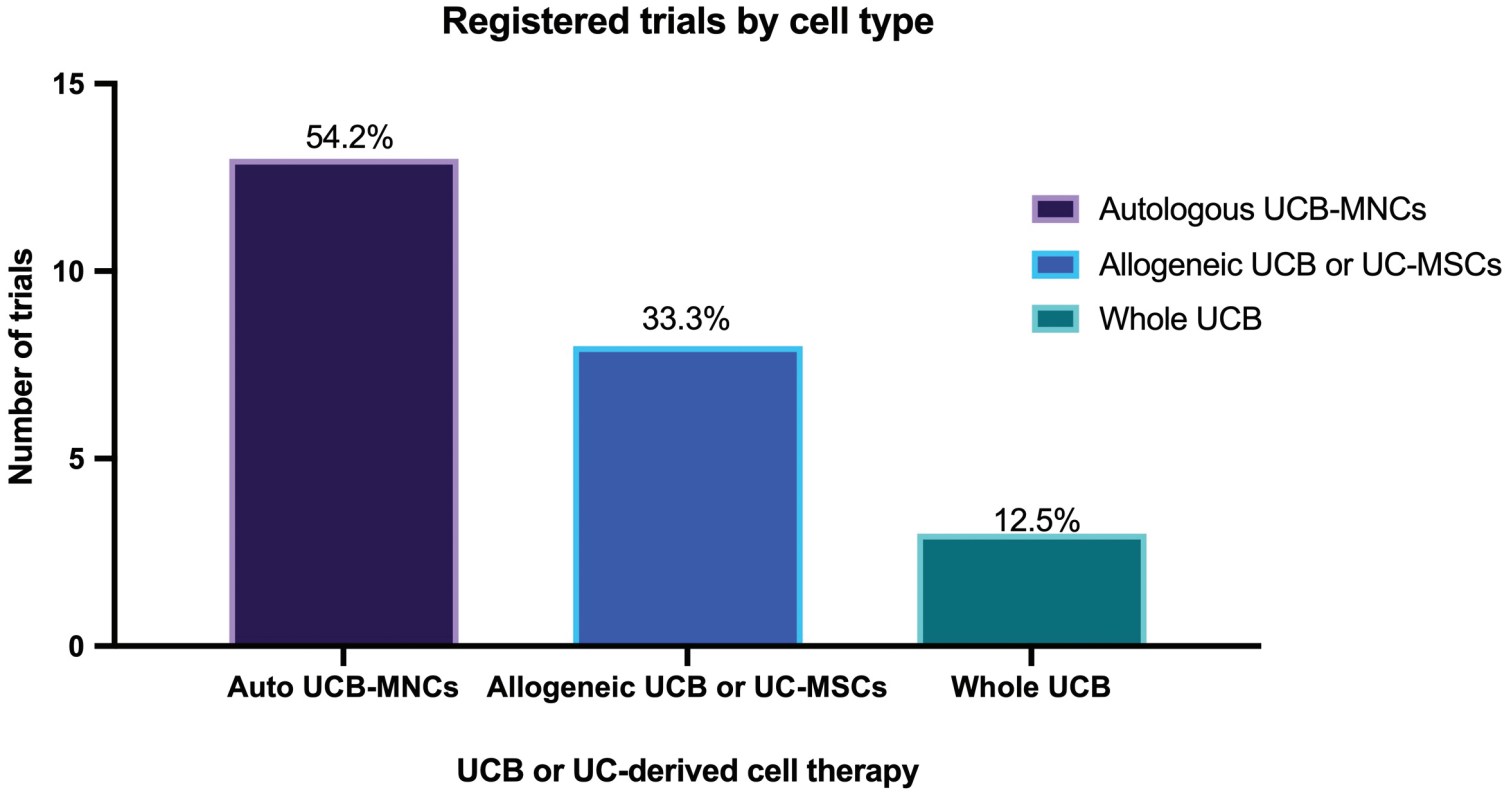
Registered, not completed trials by UCB and UC-derived cell type delivered. Abbreviations: UC, umbilical cord; UCB, umbilical cord blood.

Just over half (*n* = 13) of registered trials are administering autologous UCB-MNCs, followed by allogeneic UC-MSCs or hUCB-MSCs (*n* = 8), and whole UCB (*n* = 2). Registered UCB cell therapy trials by disease target and cell type delivered are displayed in Figs. 3 and 4, respectively.

## Review of Published Clinical Trials of UCB and UC-Derived Cell Therapy for Neonatal Morbidities and Rationale Behind Their Use

### UCB and UC-Derived MSCs for Bronchopulmonary Dysplasia

Bronchopulmonary dysplasia (BPD) is a major neonatal morbidity occurring largely in very preterm infants (born <32 weeks), affecting up to 60% of infants in that cohort.^2^ BPD arises due to a complex interaction of antenatal and neonatal factors leading to inflammation and arrest of normal lung development.^33^ Inflammation influencing development of BPD is multifactorial, contributed to by perinatal factors such as chorioamnionitis,^34^ and postnatal lung injury from mechanical ventilation.^35^ Abnormal pulmonary vascular development is a key contributor to disease, with abnormal angiogenesis during late alveolar development related to disruption in normal expression of angiogenic growth factors.^36^ This pathophysiological basis behind BPD—inflammation and angiogenesis—make it an important target to UCB and UC-derived cell therapies.

There are 3 published trials that administered hUCB-MSC therapy to neonates with BPD as a disease target, and one giving UCB-MNCs with respiratory morbidity as a primary outcome. Chang et al were the first to administer a hUCB-MSC therapy for BPD, conducting an open-label, phase I dose-escalation study of allogeneic hUCB-MSCs in 9 extremely preterm infants in South Korea.^37^ Cells were delivered via the intratracheal route at a dose of 1 or 2 × 10^7^ cells between days 7 and 14 post-birth, with a primary outcome of safety until term-corrected age. Transplantation was well tolerated, no serious adverse events were recorded, and reduced levels of pro-inflammatory cytokines, such as IL-6 and TNF-α in tracheal aspirates were noted post-intervention compared to baseline. This method was replicated by Powell et al in the US in an open-label, phase I dose-escalation study in 12 extremely preterm infants,^38^ also demonstrating no serious adverse effects using the same cell type (hUCB-MSCs) administered on days 5-14 post-birth. The Korean group have now conducted the first phase II RCT of hUCB-MSCs for BPD, administering 1 × 10^7^ cells via the intratracheal route to 33 extremely preterm infants, compared with 33 placebo controls. The primary outcome was death or moderate-severe BPD, and while no significant difference was found between the 2 groups in the primary outcome, the most pertinent finding from this trial was a significant reduction in severe BPD (19% treatment group vs 53% control) in the most extremely preterm infants at 23-24 weeks on subgroup analysis.^39^ UCB-MNCs have also been administered intravenously to 15 preterm infants (mean gestational age 31 weeks) in a study by Ren et al, an open-label, phase I placebo-control trial administering 5 × 10^7^ autologous cells on day 1 post-birth. They found no significant difference in a primary outcome of preterm morbidity or death but found the reduced duration of mechanical ventilation in the treated group compared to controls.^40^

These early-phase clinical trials are built upon pre-clinical data to suggest UCB and UC-derived MSCs work in infants at risk of BPD by attenuating inflammation that is central in its pathogenesis. Animal models where hUCB-MSCs were delivered via the trachea to neonatal rat pups demonstrated attenuation of hyperoxia-induced lung injury,^41^ modeling the cascade of inflammation that occurs following ventilation of preterm infants. In such experiments, the administration of hUCB-MSCs reduced pulmonary inflammation as demonstrated by histopathology showing reduced neutrophil infiltration and subsequent fibroblast proliferation and lung fibrosis. UC-MSCs cells may also attenuate development of BPD by secretion of VEGF,^42^ secretion of extracellular vesicles containing anti-inflammatory cytokines and growth factors, and suppress pro-inflammatory mediators, such as TNF-α and IL-6.^43,44^

The above clinical studies have demonstrated the safety and feasibility of intratracheal hUCB-MSCs and intravenous UCB-MNC therapy in small cohorts of preterm infants. Early signals of efficacy in the most extremely preterm infants are encouraging, and 8 further studies are underway, including a larger phase-2 RCT (NCT03392467).

### UCB-MNCs for Congenital Heart Disease

Congenital heart disease (CHD) affects ~1% of all live births,^45^ and while survival has improved over the last 50 years, the spectrum of CHD is still responsible for more than 250 000 child deaths each year.^46^ CHD occurs when there is abnormal development of the heart and great vessels and broadly incorporates structural deficits. This leads to abnormal circulation and strain on the myocardium, leading to eventual cardiac failure and death. The most severe lesions include single-ventricle pathologies, such as hypoplastic left heart syndrome, where infants are at risk of cardiac failure and death even with staged surgical repair.^47^ Accordingly, CHD is a disease target for UCB cell therapies, where it is suggested that UCB cells act to support an already strained or injured myocardium.^48^ Burkhart et al published an open-label, phase I trial of autologous UCB-MNCs for infants with hypoplastic left heart syndrome.^49^ Ten infants received this intervention, with 1-3 × 10^6^ cells injected directly into the myocardium during surgery, with no serious adverse effects related to the therapy reported. Participants were followed up for 6 months, with 7 completing follow-up compared to 17 historical controls. Follow-up included growth and echocardiographic parameters, and treated infants had significant improvement in body weight, right ventricular fractional area change, and ejection fraction when compared to controls.^50^

The suggested mechanism supporting an improvement with UCB therapy for CHD is via paracrine effects, relating to stimulation of vascular growth and tissue regeneration through secretion of growth factors, such as VEGF. Small animal models of cardiac dysfunction have shown increased capillary density in injured myocardium following administration of human UCB cells, suggesting the cells have angiogenic effects,^51^ and increased circulation of VEGF following UCB administration in a rodent model of dilated cardiomyopathy.^52^ This suggested benefit has been observed in large animal models of right ventricular dysfunction, where human UCB cells were administered to neonatal pigs with pulmonary artery banding, and subjects who received UCB cells demonstrated augmented right ventricular function compared to controls.^53^ A similar study administered UCB-MNCs in a porcine model of right ventricular overload, showed improved diastolic function for the cell-treated group compared to controls.^54^ Further clinical trials are ongoing to examine UCB cell therapy for infants with CHD, with 3 currently registered and recruiting (NCT03431480, NCT03558269, NCT03779711).

### UCB and UC-Derived Cells for Perinatal Brain Injury

Prevention and treatment of brain injury in neonates represents a strong area interest for UCB and UC-derived cell therapies, with focus on hypoxic ischemic encephalopathy (HIE) and preterm white matter injury representing almost half of registered, ongoing trials (Fig. 3).

HIE occurs when the brain is exposed to hypoxia during the perinatal period; this results in cellular necrosis due to primary energy failure, but also apoptosis of neural cells subsequent to excitotoxicity, oxidative stress, and inflammation.^55^ The only intervention for HIE with proven efficacy is therapeutic hypothermia. In a systematic review of clinical trials, therapeutic hypothermia reduces mortality for term infants with HIE,^56^ but there remains a high prevalence of neurodevelopmental impairment among survivors,^57,58^ driving the need for further neuroprotective interventions. Therapeutic hypothermia is not recommended for infants born at <35 weeks,^59^ thus preterm brain injury, including intraventricular hemorrhage (IVH), periventricular leukomalacia (PVL), and white matter injury of prematurity remain leading causes of lifelong neuro-disability for survivors of preterm birth,^58^ with no currently available treatments.

In 2014, Cotten et al published an open-label, phase I safety and feasibility trial of autologous UCB-MNCs in 23 term infants undergoing therapeutic hypothermia for HIE.^60^ These infants all received intravenous cells at a dose of 1-5 × 10^7^/kg at up to 4 doses (12-, 24-, 48-, and 72-hour post-birth, during the period of hypothermia), with half receiving all 4 doses. The primary outcome was feasibility and safety until 12-month follow-up, and up until that time point, no serious adverse events were attributed to the therapy. There was a hint of efficacy with 74% of cell recipients versus 41% of cooled controls having Bayley-III scores >85 in 3 domains; however, this did not reach statistical significance. A similar protocol was replicated by Tsuji et al in Japan, administering autologous UCB-MNCs at a dose of 1.4-10.9 × 10^8^ intravenously at 24, 48, and 72 hours, also during hypothermia.^61^ No serious adverse events were reported, and all infants were alive at 18 months, but outcomes were not matched to contemporary or historical controls for comparison. A third study administered autologous UCB-MNCs to two term infants with HIE in Singapore, at a dose of 6 × 10^6^ cells/kg in the first 72 hours of life, with no serious adverse effects reported.^62^ In addition to UCB-MNCs, Cotten et al have administered allogeneic UC-MSCs in 6 term infants with HIE at a dose of 2 × 10^6^/kg at 48 hours and 2 months, with no serious adverse events reported.^63^

Studies using UCB and UC-derived cell therapy for IVH and congenital hydrocephalus have also been published. Anh et al conducted an open-label, phase I dose-escalation study in 9 extremely preterm infants with IVH.^64^ They administered allogeneic hUCB-MSCs via intraventricular injection at a dose of 1-5 × 10^6^/kg, with a primary outcome of safety until term-corrected age. The procedure was well tolerated; no significant adverse effects were reported by term-corrected age, but they were not able to assess efficacy, with no matched or historical control group. Sun et al conducted an open-label, phase I trial of autologous UCB-MNCs for congenital hydrocephalus.^65^ The cells were administered in 2-4 intravenous doses of 1-5 × 10^7^/kg, at variable time points from day 6 post-birth to 4.5 years (median 2 months). The primary outcome of this study was safety and feasibility, with no serious adverse effects reported at 12 months.

Neuroprotective actions of UCB and UC-derived cells have been elucidated from pre-clinical animal data. The proposed mechanisms for neuroprotection in these studies is amelioration of secondary injury following hypoxia-ischemia and inflammation; it is likely UCB cells reduce the pro-inflammatory cytokine response (notably IL-1 and TNF-α) and subsequently reduce microglial activation and neuronal apoptosis, as evidenced by a decrease in activated caspase-3 in the brains of UCB treated subjects, and reduce oxidative stress either by a direct mechanism or as a consequence of anti-inflammatory actions. Examples of this pre-clinical evidence include rodent models of hypoxic brain injury where administration of human UCB-derived cells resulted in reduced neuronal injury,^7,66^ and in a rabbit model of hypoxia-ischemia induced cerebral palsy where UCB-treated animals showed improved motor outcomes compared to controls.^67^ This has been replicated in a large animal (lamb) model of birth asphyxia; Aridas et al showed that UCB-MNC administration 12 hours after birth improved neonatal brain metabolism on magnetic resonance spectroscopy and reduced neuro-inflammation, astrogliosis, and neuronal apoptosis, compared to untreated lambs.^68^ Isolated UC-MSCs have also been assessed, demonstrating neuroprotection in animal models of preterm hypoxia-ischemia,^69^ IVH,^70^ and neonatal stroke.^71^

In addition to hypoxia-ischemia, UCB and UC-derived cells have been shown to be neuroprotective in animal models of preterm white matter injury. Paton et al demonstrated that administration of human UCB-MNCs attenuated white matter inflammation and cell death after exposure to lipopolysaccharide (from gram-negative bacteria) in fetal sheep,^72^ representing the neuro-inflammation experienced by preterm infants affected by sepsis or chorioamnionitis. The same group also compared the effects of UCB-MNCs and UC-MSCs in the same model and discovered that UC-MSCs have a greater capacity to reduce brain inflammation, whereas UCB-MNCs demonstrated a greater neuroprotective benefit for developing white matter.^73^

The aforementioned clinical studies have demonstrated early safety and feasibility for both autologous UCB-MNCs and allogeneic UC-MSCs for perinatal neuroprotection, but there has not yet been a measure of efficacy other than the suggestion of improved outcomes in the Cotten et al’s study.^60^ There are 12 ongoing registered clinical trials studying UCB and UC-derived cell therapy for perinatal brain injury; 9 of these are targeting term HIE (including one combined with congenital diaphragmatic hernia NCT03526588), and 2 preterm brain injury, including our study of autologous UCB cells in extremely preterm infants (ACTRN12619001637134). Two of the studies are assessing for efficacy as well as safety and feasibility—a phase II study of hUCB-MSCs for IVH (NCT02890953) and a phase II study of autologous UCB-MNCs for HIE (ChiCTR-TRC-10000922).

## Challenges and Future Directions of UCB and UC-Derived Cell Therapy for Neonatal Morbidities

As we have shown in this review, UCB and UC-derived cell therapies are in early phase clinical trials for neonatal morbidities, built on strong pre-clinical evidence. Clinical outcomes to date demonstrate early safety and feasibility using a variety of UCB and UC-derived cell types, routes of administration, and disease targets. Several of the currently registered trials are attempting to assess dose-response and efficacy, and to date, just 1 RCT has been published showing early efficacy of hUCB-MSC cell therapy for BPD in extremely preterm infants.^39^

UCB and UC-derived cell therapy represents an attractive prospect in regenerative medicine due to ease of access, low immunogenicity, and well-established collection and storage processes.^8,9,12^ However, there remain questions before these cells can be established as a feasible and effective neonatal cell therapy; including volume of autologous UCB collection, particularly in the preterm population, choice of UCB and UC-derived cell therapy, allogeneic transplantation and matching, expansion of UCB-derived cell populations, and optimal timing of administration.

### Feasibility of Autologous Collection

Autologous UCB collection is desirable due to negation of concerns about engraftment and graft versus host disease (GVHD), although this is less of a concern than for other cell therapies due to the low immunogenicity of UCB derived cells. However, the quantity of UCB cells available and their dose-dependent efficacy remains to be confirmed. For autologous UCB cell therapies to be effective, they would have to demonstrate efficacy within a limited cell dose range due to the finite cell number per infant. In a recent study by Segler et al, UCB collection was determined to be feasible in high-risk infants, including preterm infants born at <30-week gestational age.^14^ They documented 74% of UCB collections contained enough viable cells for potential therapy (defined as >1 × 10^7^/kg and viability >75%); however, volumes were insufficient in a significant proportion, and collection volume decreased as gestational age decreased, with 42% of samples insufficient at <30 weeks. Similarly, in our published protocol for autologous UCB collection and re-infusion in extremely preterm infants, we have suggested a minimum volume of 9 mL to be feasible for collection and re-infusion of adequate viable cells.^74^ Studies in children have suggested an increased benefit with a higher cell dose, and dosing at a younger age. For example, an RCT of autologous UCB-MNCs for young children with cerebral palsy demonstrated improved motor function for those receiving the higher dose of 20 × 10^6^ cells/kg versus 10 × 10^6^ cells/kg.^75^ With regard to timing of treatment, in an RCT of UCB-MNCs for children with cerebral palsy, those who received cells at <36 months appeared to show increased benefit compared to older children when enrolled after 36 months.^76^ These trials hint at subgroups with greater benefit, which is why further studies are required to understand the viable cell yield in the most high-risk populations, such as extremely preterm infants, and the subpopulations likely to benefit the most.

### UCB Cell Expansion and/or Allogeneic Transplantation

Due to the challenge of potentially insufficient cell counts for very small preterm neonates, an expanded allogeneic or autologous product may be required, paving the way for multiple doses if shown to be effective. When it comes to an allogeneic product, transplantation of allogeneic UCB-MNCs has not yet been trialed in neonates, while allogeneic UC-MSCs have been given intravenously.^63^ Transplantation of allogeneic UCB-MNCs from HLA-matched siblings for children with established cerebral palsy is being trialed, without conditioning or immune suppression of patients,^77^ and a recent review has demonstrated reassuring safety for this practice in children.^78^ However, use of an allogeneic UCB-MNC product introduces risk of GVHD due to engraftment and proliferation of donor T cells; this has been documented following transfusion in the neonatal population,^79,80^ and is the reason neonatal blood products are routinely irradiated, particularly for those born prematurely.

With regard to expansion, expanded allogeneic cell products are already being used in studies administering hUCB and UC-derived MSCs, as they are proven to be isolated and expanded in sufficient quantities for completed and ongoing phase I and II trials, and safely administered to 69 neonates thus far^37-39^ without signs of engraftment. Expansion of other UCB cell lines, such as HSCs and EPCs is an ongoing area of research. CD34^+^ UCB cells have been successfully cultured and expanded in pre-clinical studies^81-83^ and in clinical studies in adult hematological malignancies,^84^ but there are no clinical trials testing the use of an expanded CD34^+^ product in neonates. The effects of CD34^+^ and EPCs are being investigated in pre-clinical studies^85^; however, the majority of these studies are not using EPCs derived from UCB, and progression to clinical trials has so far only been for adult diseases.^86^ Further pre-clinical studies of expanded UCB cells in small and large animal models are underway and will be crucial in the development of a potential expanded UCB cell therapy.

### Timing of Cell Delivery

Pre-clinical studies have shown timing of UCB-derived cell administration is important. For example, Li et al showed a difference in the effects of UCB cell administration at 12 hours versus 5 days in an ovine model of preterm hypoxia-ischemia, wherein early administration showed increased neuroprotection, with the suggested mechanism being anti-inflammatory and anti-oxidative effects of the UCB-MNCs occurring at key time points in the cascade of neurological injury.^87^ A further study examined the role of multiple UCB cell dosing in a rat model of hypoxia-ischemia, reporting increased benefit from multiple versus single dosing.^88^ These studies provide evidence that timing and repeat dosing are important factors to consider when attempting to ameliorate secondary injury in morbidities influenced by inflammation.

## Conclusions

Pre-clinical evidence supports the anti-inflammatory and regenerative effects of UCB and UC-derived cell therapies for the neonatal lungs, heart, and brain, and this knowledge has been translated safely in 11 early-phase trials and 1 phase II RCT.

Safety and feasibility of intravenous UCB and UC-derived cell therapy has been established in infants with term HIE (37 participants), and in preterm infants with BPD (54 participants). Studies to date have been heterogeneous with respect to UCB or UC-derived cell type, dose, and timing of administration.

Early signals of efficacy are present for hUCB-MSCs in extremely preterm infants for BPD and UCB-MNCs for HIE and hypoplastic left heart. Further clinical trials are required to test safety, feasibility, and early efficacy by selecting disease as a primary outcome across different cell types, including timing of administration and dose effect. Then, collaboration between cell therapy groups and those working in neonatal intensive care will be required to establish multicenter RCTs to test the efficacy of UCB and UC-derived cell therapies in neonates.

## Supplementary Material

szab024_suppl_Supplementary_Appendix

## Data Availability

No new data were generated or analyzed in support of this research.
