# Supplementary material for: Umbilical Cord Blood and Cord Tissue-Derived Cell Therapies for Neonatal Morbidities: Current Status and Future Challenges
Source: Stem Cells Transl Med. 2022 Mar 8;11(2):135–45. doi: 10.1093/stcltm/szab024 (PMC8929446; doi:10.1093/stcltm/szab024)
Supplement: szab024_suppl_Supplementary_Appendix [file szab024_suppl_supplementary_appendix.pdf]

Appendix 1: PRISMA diagram detailing search process

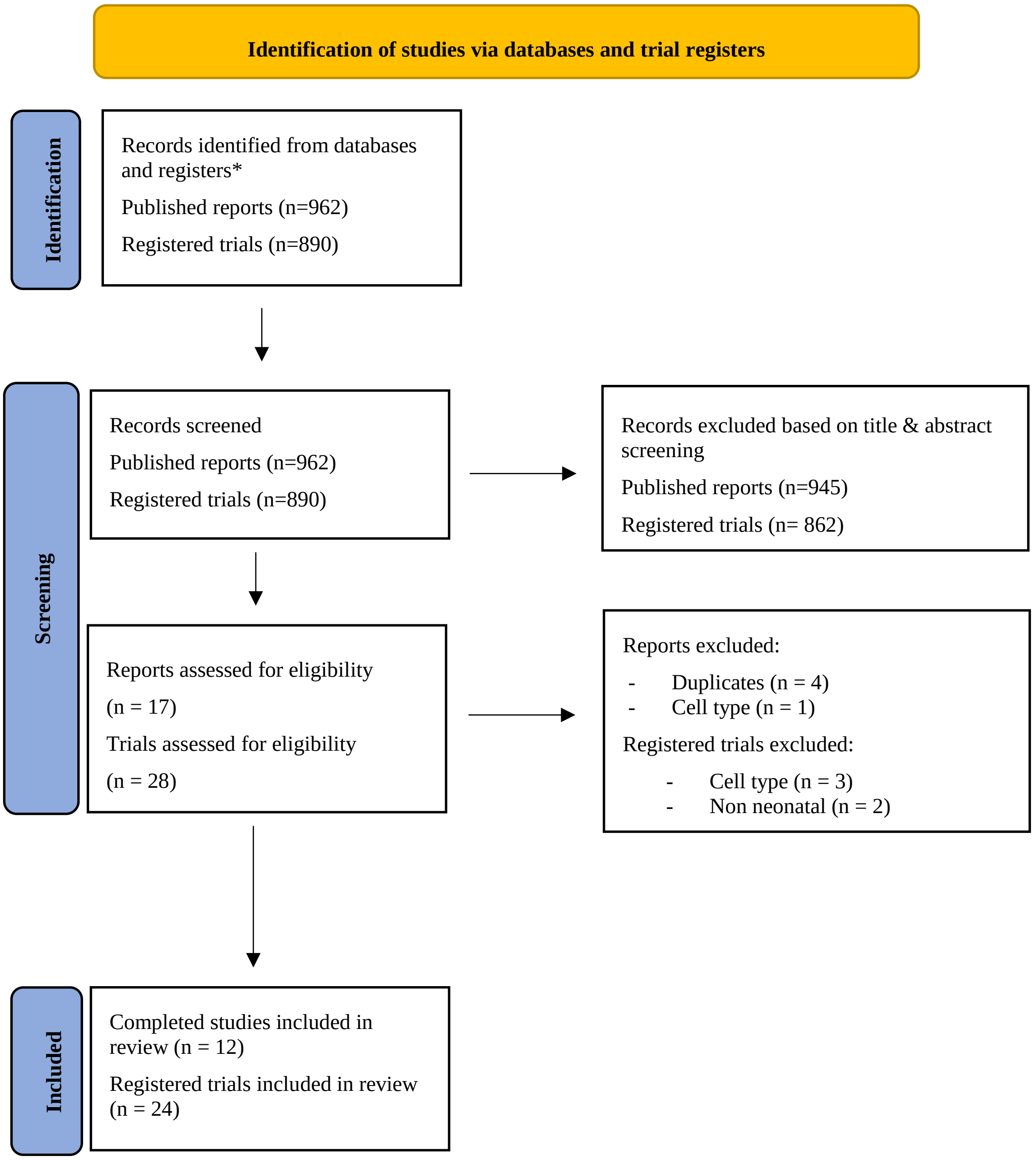

*Databases: PubMed, Ovid Medline, Google Scholar

*Registers: ClinicalTrials.gov, ANZCTR.org.au, chictr.org.cn, clinicaltrialsregister.eu, celltrials.org
